# Novel Gross Deletion Mutations in *NTRK1* Gene Associated With Congenital Insensitivity to Pain With Anhidrosis

**DOI:** 10.3389/fped.2021.638190

**Published:** 2021-03-04

**Authors:** Lulu Li, Chao Jia, Yue Tang, Yuanyuan Kong, Yaofang Xia, Li Ma

**Affiliations:** ^1^Department of Newborn Screening Center, Beijing Obstetrics and Gynecology Hospital, Capital Medical University, Beijing Maternal and Child Health Care Hospital, Beijing, China; ^2^Department of Neonatology, Hebei Provincial Children's Hospital, Shijiazhuang, China

**Keywords:** Chinese families, HSAN, whole exome sequencing, mutations, CIPA, NTRK1

## Abstract

**Background:** Congenital insensitivity to pain with anhidrosis (CIPA) is a rare inherited autosomal recessive disorder characterized by insensitivity to noxious stimuli, anhidrosis, recurrent fever, and intellectual disability. CIPA is mainly caused by mutations in the neurotrophic tyrosine kinase receptor type 1 gene (*NTRK1*). This study aims to identify pathogenic mutations underlying CIPA in two unrelated Chinese families.

**Methods:** DNA was extracted from blood samples of patients and their available family members and subjected to whole exome sequencing (WES). Real-time PCR (qPCR), Gap-PCR, and Sanger sequencing were applied to verify the identified variants.

**Result:** We found novel compound gross deletion mutations [exon1-6 del (g.1-1258_10169del); exon5-7 del (g.6995_11999del)] of *NTRK1* (MIM 191315) gene in family 1 and the compound heterozygous mutations [c.851-33T>A; exon5-7 del (g.6995_11999del)] in family 2. Interestingly, we discovered the intragenic novel gross deletion [exon5-7 del (g.6995_11999del)] mediated by recombination between Alu elements.

**Conclusions:** The present study highlights two rare gross deletion mutations in the *NTRK1* gene associated with CIPA in two unrelated Chinese families. The deletion of exon1-6 (g.1-1258_10169del) is thought to be the largest *NTRK1* deletion reported to date. Our findings expand the mutation spectrum of *NTRK1* mutations in the Chinese and could be useful for prenatal interventions and more precise pharmacological treatments to patients. WES conducted in our study is a convenient and useful tool for clinical diagnosis of CIPA and other associated disorders.

## Introduction

Congenital insensitivity to pain with anhidrosis (CIPA; MIM 256800) or hereditary sensory and autonomic neuropathy type IV (HSAN-IV) is a rare autosomal recessive disease characterized by a loss of pain sensation, anhidrosis (inability to sweat), irregular body temperature, growth retardation, and progressive central nervous system defects (1–3). The HSAN affects both sexes and has been classified into five distinct types proposed by Dyck and Ohta (4). These are sensory radicular neuropathy type I (HSAN I), congenital sensory neuropathy type II (HSAN II), familial dysautonomia (FD III), congenital insensitivity to pain with anhidrosis type IV (HSAN IV), and congenital indifference to pain associated with intellectual disability type V (HSAN V) (5). Each HSAN disorder is caused by different genetic factors that affect specific aspects of small fiber neurodevelopment, which results in variable phenotypic expression, and it is predominantly inherited as an autosomal recessive and dominant manner (6). HSAN type I is caused by the *SPTLC1* gene located on chromosomes 9q22.1–22.3, inherited as autosomal dominant form (7). However, HSAN type-II, HSAN type-III, HSAN type-IV, and HSAN type-V are inherited in an autosomal recessive manner caused by different genetic factors (4, 5).

CIPA was first described in 1963 by Swanson (8). It is caused by recessive loss-of-function mutations in neurotrophic tyrosine kinase receptor type 1 gene (*NTRK1*; MIM 191315) (9, 10). The gene *NTRK1* encodes tropomyosin receptor kinase A (TrkA) protein, which has a high affinity for nerve growth factor (NGF) receptor (11). NGF/TrkA signal is affected by *NTRK1* gene mutation (12). In recent years, with the enrichment of clinical experience and the development of sequencing technology, numerous CIPA cases have been reported, having homozygous or compound heterozygous mutations in *NTRK1* (13–15).

Here, we describe two unrelated Chinese families having HSAN type IV (CIPA) phenotype. Using whole exome sequencing (WES), we identified novel gross deletion mutations [exon1-6 del(g.1-1258_10169del) and exon5-7 del (g.6995_11999del)] in the *NTRK1* gene.

## Materials and Methods

### Ethical Sight

**Figure 1:**
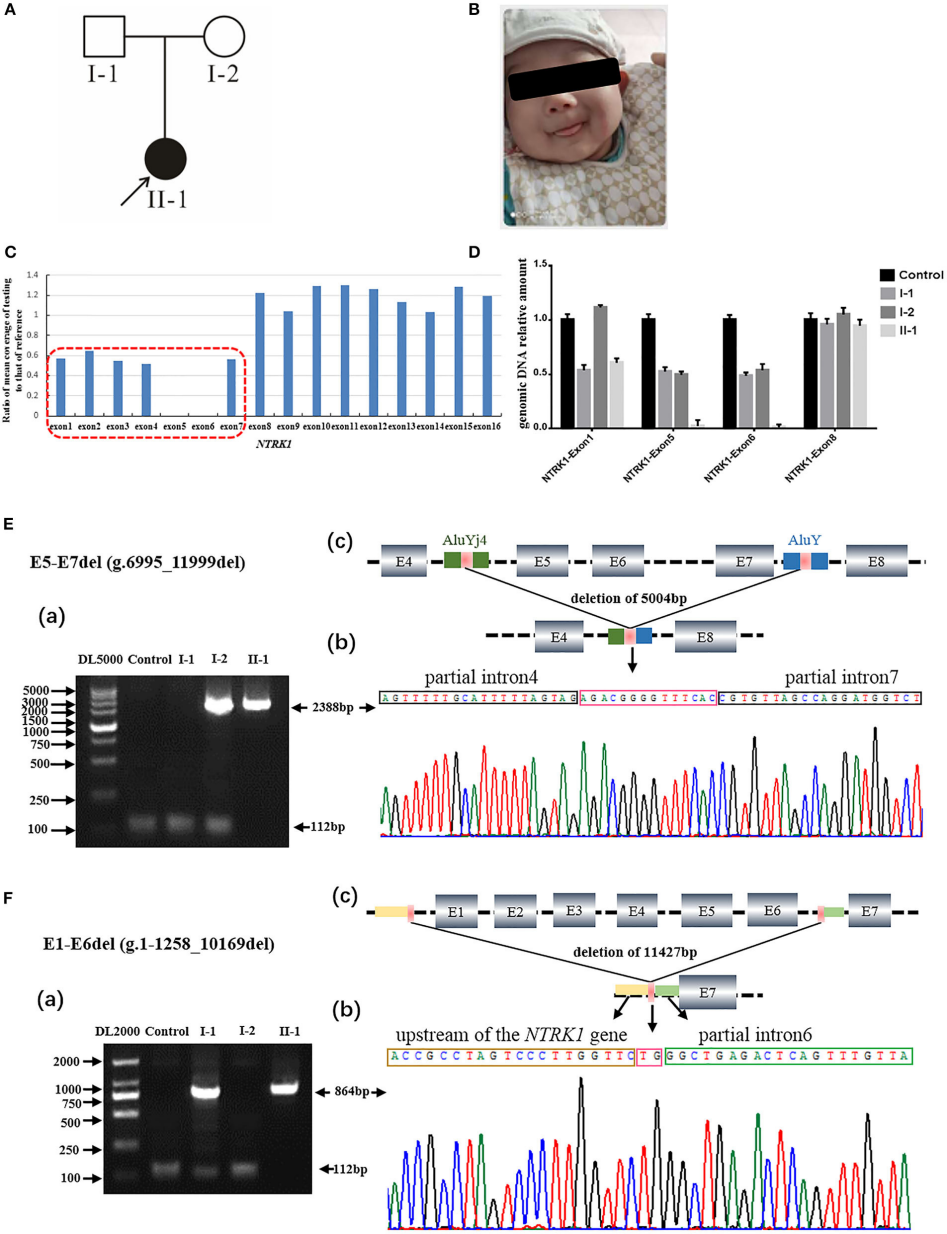
Identification of compound heterozygous gross deletion mutations of neurotrophic tyrosine kinase receptor type 1 (*NTRK1)* in family 1. **(A)** Pedigree chart of family1, black arrow indicates the proband. **(B)** Current situation of patient 1. **(C)** The result of whole exome sequencing (WES), the blue bar chart Means coverage of each exon of *NTRK1* gene. Bar chart of readcount showing a suspicious area of CNVs (red squares) of *NTRK1*. It indicates the mutation conclude deletion of exon1-6 and the deletion of exon6-7. **(D)** The real-time quantitative PCR result (right) verified the compound heterozygous deletion. Deletion of exon1-6 derived from the father and deletion of exon6-7 derived from the mother. **(E)** Identification of the gross deletion E6-E7del (g.6995_11999del): (a) Gap-PCR products about 2,388 bp, the forward primer in exon4 and the reverse primer in exon8, proband and his mother amplified the products. Except for the patient 1, the other three people could amplify the internal reference fragment (112 bp); (b) DNA sequencing of the Gap-PCR products unveiled a deletion of 5,004 bp, and the breakpoint junction was located within two Alu repetitive elements with 14 bp common fusion; (c) The schematic map of gross deletion: E5-E7del (g.6995_11999del), gross deletion covering exons5, exon6, exon7, and introns in *NTRK1*. **(F)** Identification of the gross deletion E1-E6del (g.1-1258_10169del): (a) Gap-PCR products about 864 bp, the forward primer in upstream of the *NTRK1* gene, and the reverse primer in exon7, proband and his father amplified the products. The internal reference fragment (112 bp) only found between the control and the parents; (b) DNA sequencing of the Gap-PCR products unveiled a deletion of 11,427 bp, and the breakpoint junction with 2 bp common fusion; (c) The schematic map of gross deletion:E1-E6del (g.1-1258_10169del) gross deletion covering exon1-6, upstream of the *NTRK1* gene, and introns in *NTRK1*.

**Figure 2:**
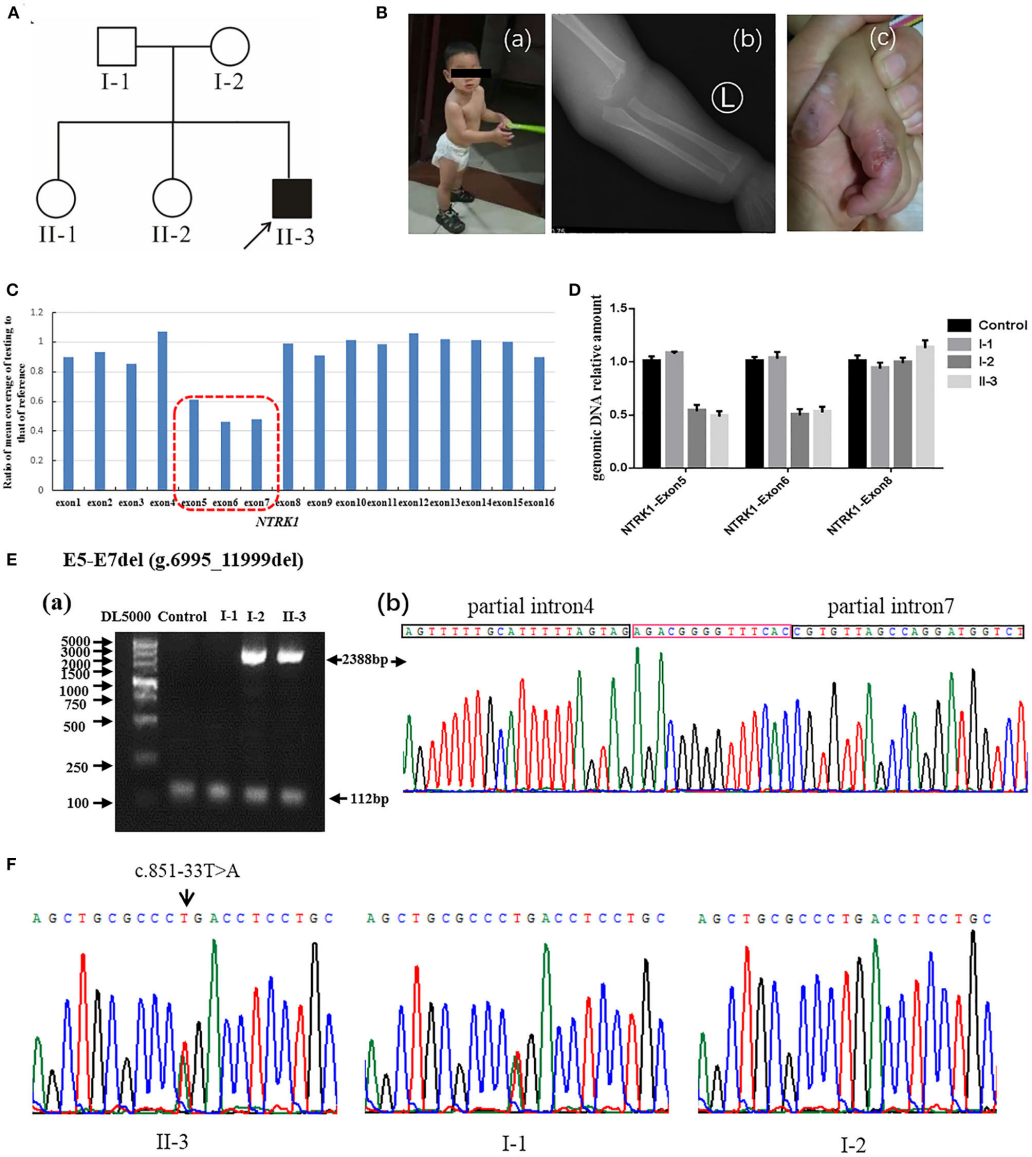
Identification of compound heterozygous mutations of *NTRK1* in family 2. **(A)** Pedigree chart of family 2, black arrow indicates the proband. **(B)** The clinical manifestations of the proband: (a) current situation of patient 2 (b) damaged hand; (c) X-ray image showing a humeral fracture. **(C)** The result of whole exome sequencing (WES), the blue bar chart means coverage of each exon of *NTRK1* gene. Bar chart of readcount showing a suspicious area of CNVs (red squares) of *NTRK1*. It indicates the heterozygous gross deletion mutation from exon5 to exon7. **(D)** The real-time quantitative PCR result verified the gross deletion, and the heterozygous mutation of deletion derived from the mother. **(E)** Identification of the gross deletion: E6-E7del (g.6995_11999del): (a) Gap-PCR products about 2,388 bp, proband, and his mother amplified the products; the internal reference fragments (112 bp) can be amplified in all samples; (b) DNA sequencing of the Gap-PCR products, the breakpoint junction was located within intron4 and intron7. **(F)** Gene sequencing results: the proband carries heterozygous splicing mutation of *NTRK1* gene c.851-33T>A, and the mutation derived from the father.

The study design and protocol was approved by the Ethical Review Committee (ERC) Department of Newborn Screening Center, Beijing Obstetrics and Gynecology Hospital, Capital Medical University. Signed informed consent for the genetic analysis and publication of data was obtained from the patient's legal guardians. Pedigree was drawn (Figures 1A, 2A), and the affected individuals were thoroughly examined by a local geneticist and physiologist.

### Blood Sample Collection and DNA Extraction

Fresh blood sample was drawn from the affected and normal individuals. Genomic DNA was extracted using phenol chloroform method and was quantified by Nanodrop-2000 spectrophotometer.

### Whole Exome Sequencing and Data Analysis

The two probands were subjected to whole exome sequencing (WES) at Beijing MyGenostics Technology Co., Ltd. WES libraries were prepared following the manufacturer's recommendations. Paired-end sequencing was performed on a NextSeq 500 sequencer (Illumina, San Diego, CA). After sequencing, the raw data were saved as a fastq format. After quality control, the clean reads were mapped to the UCSC hg19 human reference genome using BWA(0.7.12) software (http://bio-bwa.sourceforge.net/). The fastq file was converted to the bam file, and then to vcf file. The ANNOVAR software (http://annovar.openbioinformatics.org/en/latest/) was used to annotate the variants (Supplementary Table 1). We mainly focused on protein-altering variants such as missense, non-sense, splice site variants, and coding indels, with alternative allele frequencies <0.005 in the Exome Variant Server (EVS, https://evs.gs.washington.edu/EVS/), Genome Aggregation Database (gnomAD https://gnomad.broadinstitute.org/), 1000 Genomes (http://www.1000genomes.org/), dbSNP (http://www.ncbi.nlm.nih.gov/SNP/), and in the Exome Aggregation Consortium (ExAC, http://www.exac.broadinstitute.org) and an internal exome database including ~200 exomes (16). To identify potential causal variants, we further filtered the variants based on a recessive and dominant mode of inheritance. Different bioinformatics software including Mutation Taster (http://www.mutationtaster.org/), Polyphen-2 (http://genetics.bwh.harvard.edu/pph2), and Sorting Intolerant From Tolerant (SIFT, http://www.sift.jcvi.org/) were used for functional effect prediction. The Human Gene Mutation Database (HGMD; http://www.hgmd.cf.ac.uk/ac/index.php) was used to search for identified variant novelty. Finally, in the assessment of variant interpretations and pathogenicity, the American College of Medical Genetics and Genomics (ACMG) 2015 guidelines were used (17).

### CNV Analysis

The CNV analysis was performed at Beijing MyGenostics Technology Co., Ltd. The Cnvkit software (https://cnvkit.readthedocs.io/en/stable/index.html) was used to analyze the samples captured in the same pool. The read depth of target region and non-target region was counted, and then the depth was homogenized and corrected in the sample, and compared with the control set, and the copy number information was obtained. Multiple samples to establish reference for error correction of reads-depth were used. Discrete copy number fragments were calculated through the built-in segmentation algorithm of the software. A ratio below 0.7 was speculated as putative deletions, and the ratio that rose above 1.3 was considered as putative duplications (18).

### Real-Time Quantitative Polymerase Chain Reaction

The genomic DNA or cDNA reference sequences of *NTRK1* (hg19, NC_000001.10 and NM_001012331.1) were obtained from the University of California, Santa Cruz (UCSC) Genome browser database (https://genome.ucsc.edu/). Primer 5.0 primer software was used to design the specific PCR primers (Supplementary Table 2). Real-time quantitative PCR (Q-PCR) reaction was carried out using the CFX96 Touch Real-Time PCR Detection System (Bio-Rad, Hercules, CA, USA). SYBR Green I (TaKaRa) was used as the fluorescent label. The total reaction system is a 10-μl final volume; each assay was performed in quadruplicate. The reactions were carried out by the following program: 95°C for 30 s followed by 40 cycles of 95°C for 10 s, 62°C for 15 s, and 72°C for 20 s.

### Mutation Validation

Primer 5.0 primer software was used to design the Gap-PCR primers and internal reference primer of exon6 in the deleted fragment (Supplementary Table 2). The PCR reaction was commenced with an initial 3-min denaturation step at 95 °C, followed by 38 cycles of denaturation (94 °C) for 30 s, annealing (60 °C) for 30 s, and extension (72 °C) for 50 s, and ended with a final extension step at 72 °C for 8 min. The PCR products were purified and subjected to Sanger sequencing using an ABI3700 automated sequencer (PE Bio systems, Foster City, CA). The Sanger sequencing results were examined and compared with the help of visual software such as Chromas Lite and Codon Code Aligner. Sanger DNA sequencing was further used to identify the break-points of gross deletions.

## Results

### Clinical Investigation

In this study, we recruited two Chinese families (family 1 and family 2) affected by CIPA due to variants *NRKT1*, which were identified by WES technology. Both families were visited at the Department of Newborn Screening Center, Beijing Obstetrics and Gynecology Hospital, Capital Medical University. Normal elders or guardians were interviewed to record the history of the disease, marriage type, affected and non-affected subjects, and disease status, and to generate the family tree (Figures 1A, 2A).

#### Family 1

The members of family 1 (Figure 1A) live in Hebei Province of China. Family 1 has 1 affected individual (II-1) having typical features of autosomal recessive CIPA (Figure 1A). The family's medical history, pregnancy, and delivery were uneventful. Parents of the affected individuals were physically and mentally normal and healthy. The proband (II-1) showed intermittent fever with abnormal body temperature. Her clinical features include loss of pain sensation, anhidrosis, irregular body temperature, and dry skin (Table 1). There was no family history of CIPA seen in this family. Other clinical features such as intelligent quotient (IQ), height, cardiac, respiratory, skeletal, and hair were observed as normal (Figure 1B).

**Table 1:** Mutations of the CIPA patients recruited in this study.

**Patient**	**Gene**	**Zygote type**	**Allele origin**	**Variant location**	**Nucleotide (amino acid) change**	**Novel variant**
1	*NTRK1*	C-het	P	E1–E6	exon1-6 del (g.1-1258_10169del)	Yes
			M	E5–E7	exon5-7 del (g.6995_11999del)	Yes
2	*NTRK1*	C-het	P	I7	c.851- 33T>A	No
			M	E5–E7	exon5-7 del (g.6995_11999del)	Yes

*C-het, compound heterozygote; P, paternal; M, maternal; E, exon; I, intron*.

#### Family 2

Family 2 (Figure 2A) also originates from Hebei Province of China. The family comprises one affected male individual (II-3), indicating an autosomal recessive inheritance. The parents married without close relatives, and there was no family history of CIPA. The patient had congenital CIPA phenotype (Figure 2B): absence of normal responses to painful stimuli, anhidrosis, and recurrent fever. Last clinical examination, at 1 year and 3 months of age (Figure 2Ba) revealed fracture of the left humerus (Figure 2Bb), self-harm and biting of hands (Figure 2Bc), peeling fingers, dry and cracked skin, and growth retardation was observed. Other anomalies such as cardiac, respiratory, skeletal, hearing anomalies were seen normal. Detailed clinical information of the affected individuals is summarized in Table 1.

### Mutation Identification and Confirmation

WES was performed for two affected individuals as described previously (16). Filtration of the identified variants was performed considering all patterns of inheritances. We focused only on pathogenic, likely pathogenic, VUS, non-synonymous (NS) variants causing missense, non-sense, frame-shift, splice site variants (SS), coding insertions, or deletions (indel). We identified gross novel deletion mutations in the two unrelated CIPA Chinese families (Figures 1C, 2C). The pathogenic variants of the proband were detected in both alleles of *NTRK1*; their parents carries one pathogenic allele (Table 1).

Q-PCR analysis showed that patient 1 and her father had the same exon1 deletion, and patient 1 carries the homozygous deletion of exon5 and exon6 (Figure 1D). The result of Q-PCR verified that patient 2 carries the heterozygous deletion of exon5 and exon6, and the mutation of gross deletion was derived from the mother (Figure 2D).

Agarose gel electrophoresis indicated that patient 1 and his mother had the same 2,388-bp products (Figure 1Ea). By gap-PCR and Sanger sequencing, we found a 5,004-bp (g.6995_11999del) deletion including exon5-7 (Figure 1Eb). We made the schematic map of this gross deletion: the breakpoints were found in introns 4 and 7, and the junction of breakpoints was located within two Alu repetitive elements, which share a 14-bp common fusion sequence (Figure 1Ec). This same gross deletion was detected in family 2 (Figure 2E). In the Sanger sequencing of the 864-bp Gap-PCR products (Figure 1Fa), we found that patient 1 and his father had the deleted 11,427-bp (g.1-1258_10169del) fragments (Figure 1Fb), and the breakpoints were found in intron 6 and upstream of *NTRK1* (Figures 1Fb,c). Meanwhile, except for patient 1, the other three people could amplify the internal reference fragment (112 bp) to verify the homozygous deletion of exon6 (Figures 1Ea,1Fa). Patient 2 carries the gross deletion mutation (g.6995_11999del) derived from the mother (Figure 2Ea; Supplementary Figure 1). Through the gel image, we can see that all samples can amplify the internal reference fragments (112 bp), confirming that patient 2 is a heterozygous carrier of the mutation (Figure 2Ea). By Sanger sequencing, we found that patient 2 carries heterozygous splicing mutation of the *NTRK1* gene c.851-33T>A and the mutation derived from the father (Figure 2F, Supplementary Figure 1).

## Discussion

The *NTRK1* is also known as *MTC, TRK, TRK1, TRKA, Trk-A*, and *p140-TrkA*, respectively. It is located on chromosome number 1q23.1 and covers about 20 kb of DNA. It has 17 exons and encodes 790/796 amino acid *NTRK1* enzymes (9, 10). The TrkA receptor has three functional domains: the extracellular domain, transmembrane domain, and intracellular tyrosine kinase domain (11, 12). To date, 128 different types of *NTRK1* gene mutations have been described in the human gene mutation database (HGMD, version Professional 2020.6; http://www.hgmd.org) causing CIPA phenotypes (Supplementary Table 3). The most reported mutations were in the intracellular tyrosine kinase domain. However, there is no clear correlation between the genotype and phenotype of CIPA patients (2, 19). Among 128 different mutations, we found that only four reports about gross deletion mutations (2, 20–23); the largest deletion range was about 1,403 bp, which included exon5-6 (Supplementary Table 3). It affects the extracellular domain (2).

In this study, we investigated two Chinese families (A and B) with CIPA. The two patients exhibited the common clinical characteristic symptoms of CIPA (24, 25): insensitivity to pain, anhidrosis, recurrent fever, and xerosis cutis, chapped (Table 2). Patient 2 have shown bone fracture, intellectual disability, and self-mutilation symptoms, but are not found in patient 1. We suspect that patient 1 is still in infancy. She has no teeth, and she does not have the ability to move independently. So some phenotypes have not yet appeared.

**Table 2:** Clinical manifestations of the CIPA patients recruited in this study.

**Patient**	**Affected patients number in family**	**Age at first visit (day)**	**Gender (M&F)**	**Insensitivity to pain**	**Anhidrosis**	**Recurrent fever**	**Bone fractures/times**	**Osteomyelitis**	**Intellectual disability**	**Self-mutilation**	**Irascibility**	**Xerosis cutis, chapped**
1	1	16d	F	Y	Y	Y	N	N	N/A	N	N/A	Y
2	1	18d	M	Y	Y	Y	Y/1	N	Y	Y	N/A	Y

*F, female; M, male; Y, yes; N, no; N/A, not available*.

In family 1, we identified compound novel gross deletion mutations [exon1-6 del (g.1-1258_10169del) and exon5-7 del (g.6995_11999del)] of the *NTRK1* gene in patient 1. Both mutations are located in the extracellular domain. To our knowledge, the mutation [exon1-6 del] is the first reported and the largest *NTRK1* deletion of *NTRK1* in CIPA. The gross deletion contains the upstream of *NTRK1* gene, so it may cause the promoter to not be recognized, and the normal transcription of the gene is affected. Mutation [exon5-7 del (g.6995_11999del)] may lead to a premature termination of translation. By checking the UCSC Genome Browser (https://genome.ucsc.edu/), we found that the breakpoint was located within Alu elements. The breakpoint in intron 4 was within the AluYj4 region (chr1:156867686-156867934); the genomic size of AluYj4 element is about 249 bp. The breakpoint in intron 7 was within the AluY (chr1:156872676-156872976) region, the genomic size of AluY element is about 301 bp.

Based on the difference in key diagnostic nucleotides, the main Alu lineages are divided into AluJ, AluS, and AluY (26). AluYj was an AluY subfamily member, and it is classified as AluYj3 and AluYj4 (27). Alu element accounts for 10% of the human genome, due to the high similarity and richness of these Alu elements, it often participates in the rearrangement of the genome, thereby affecting gene expression and leading to the occurrence of diseases (28, 29). At present, about 515 cases of Alu-mediated deletion events have been reported, mainly through Alu insertion-mediated deletion (AIMD) or Alu recombination-mediated deletion (ARMD). Among them, the ARMD event was mainly caused by the recombination of Alu elements in different subfamilies (26, 30, 31). Although the AluJ subfamily is more abundant than the AluY subfamily, the AluY subfamily has higher sequence homology. Therefore, the ARMD event is closely related to the AluY subfamily (32). By using the NCBI Blast (https://blast.ncbi.nlm.nih.gov/Blast.cgi), we found that the similarity of these two Alu elements is as high as 89%. Our researches confirm once again (2) that Alu elements may be an important reason for *NTRK1* intragenic deletions.

In family 2, affected individuals revealed compound heterozygous mutations [c.851-33T>A and exon5-7 del (g.6995_11999del)]. The mutation c.851- 33T>A has been reported repeatedly at home and abroad (33, 34), which is the most common hot spot mutation in the CIPA population in Asia c.851-33T>A was first found by Miura et al. (35). The mini-gene assay showed that the mutation resulted in the formation of a branching site, which was re-identified as an Ag signal and led to the insertion of 137 bp at the 3′ end of intron 7. The mutation exon5-7 del (g.6995_11999del) is the same as the mutation of patient 1.

Next-generation sequencing (NGS) technology includes WES, WGS, and Panel. It has expanded in the last decades with significant improvements in the reliability, sequencing chemistry, pipeline analyses, data interpretation, and costs. Such advances make the use of NGS feasible in clinical practice today for diagnostic evaluation of patients with suspected genetic disorders. Geng et al. (2) examined the *NTRK1* mutation spectrum and prevalence among 36 patients with CIPA in the Chinese population and described that there is no obvious correlation between genotype and phenotype relationship with *NTRK1* mutation. They performed NGS technology and found gross deletion and deep intronic mutation in the *NTRK1* gene (2). Zhao et al. (33) recruited 21 patients with CIPA and identified multiple forms of variants responsible for CIPA. By using WES technology, they identified gross deletion mutation in the deep intron, and they found a rough boundary of uniparental homodisomy (33). More interestingly, we found that Martin Farr et al. (36) used the NGS technology to detect the Alu-medicated exon duplication for Fabry disease.

CIPA is rare and rarely reported at home and abroad (1–3). The main clinical manifestation of CIPA is recurrent fever without obvious regularity (24). Many clinicians do not fully understand the disease and often misdiagnose it as infectious fever. At the same time, children with CIPA have lack of pain and may have self-mutilation behaviors such as tongue biting and hand biting, and are prone to accidental injuries such as fractures and burns (25). At present, there is no effective treatment for the disease, which mainly adopts cooling and active anti-infection symptomatic treatment. Therefore, high-throughput sequencing technology is used to detect and diagnose early, and effective protective treatment is carried out for children, to reduce the disability of children and ensure a better prognosis.

## Conclusion

In conclusion, we identified the pathogenic mutations in two CIPA families and found two novel gross deletion mutations. It enriches the pathogenic mechanism of the *NTRK1* gene. The data will be helpful in diagnosing and predicting CIPA, and continued study of *NTRK1* gene mutations will be valuable for identification of affected newborns or gene carriers in families with an identified mutation. WES is not only convenient and fast but also can be used for rare deep intron variation and gross deletion.

## Data Availability Statement

The datasets presented in this study can be found in online repositories. The name of the repository and accession number can be found at: National Center for Biotechnology Information (NCBI) GenBank, https://www.ncbi.nlm.nih.gov/genbank/, MW467564.

## Ethics Statement

Signed informed consent for the genetic analysis and publication of data was obtained from the patient's legal guardians.

## Author Contributions

LL and CJ performed the sequencing analysis and wrote the manuscript. YT conducted the data collection as well as data analysis. YK and YX helped with recruiting patients. LM conceived the study and supervised this research. All authors performed critical reading and approved the final version of the manuscript.

## Conflict of Interest

The authors declare that the research was conducted in the absence of any commercial or financial relationships that could be construed as a potential conflict of interest.
