# Supplementary material for: Novel Gross Deletion Mutations in NTRK1 Gene Associated With Congenital Insensitivity to Pain With Anhidrosis
Source: Front Pediatr. 2021 Mar 4;9:638190. doi: 10.3389/fped.2021.638190 (PMC7969531; doi:10.3389/fped.2021.638190)
Supplement: Supplementary file 3 [file Data_Sheet_1.DOCX]

**Supplementary Table 2. Primers used in this study.**

| **Primer name** | **Sequence (5' → 3')** | **T_m_ (°C)** | **Size (bp)** |
| --- | --- | --- | --- |
| *NTRK1*-Q1-F  *F* | GTCTTTAACACCGCCCAGC | 60 | 190 |
| *NTRK1*-Q1-R | GATGCCAGTATCAGCCAAGC |  |  |
| *NTRK1*-Q5-F  *F* | GGAGTGCCTGAACAGAAGCT | 60 | 102 |
| *NTRK1*-Q5-R | CCCTTACACCACCTCCCTCA |  |  |
| *NTRK1*-Q6-F  *F* | GAAGGTCCAGGTGCCCAAT | 60 | 112 |
| *NTRK1*-Q6-R | CTCCAGCTCTGTGAGGATCC |  |  |
| *NTRK1*-Q8-F  *F* | TCAACAACGGCAACTACACG | 60 | 104 |
| *NTRK1*-Q8-R | CTCGGGGTTGAACTCGAAAG |  |  |
| Breakpoint *-NTRK1*-Q4-F | TCCTCCCCTCCCTCATTCTG | 60 | - |
| Breakpoint-*NTRK1*-Q8-R | CTCGGGGTTGAACTCGAAAG |  |  |
| Breakpoint *-NTRK1-*up1400-F | GACATCTGCGGGGTAACTTC | 60 | - |
| Breakpoint*-NTRK1*-Q7-R | CAGGAGACGTTGACCTGAACA |  |  |
| *NTRK1*-E6-ref-F  *F* | GAAGGTCCAGGTGCCCAAT | 60 | 112 |
| *NTRK1*-E6-ref-R | CTCCAGCTCTGTGAGGATCC |  |  |

**Supplementary Figure 1.**


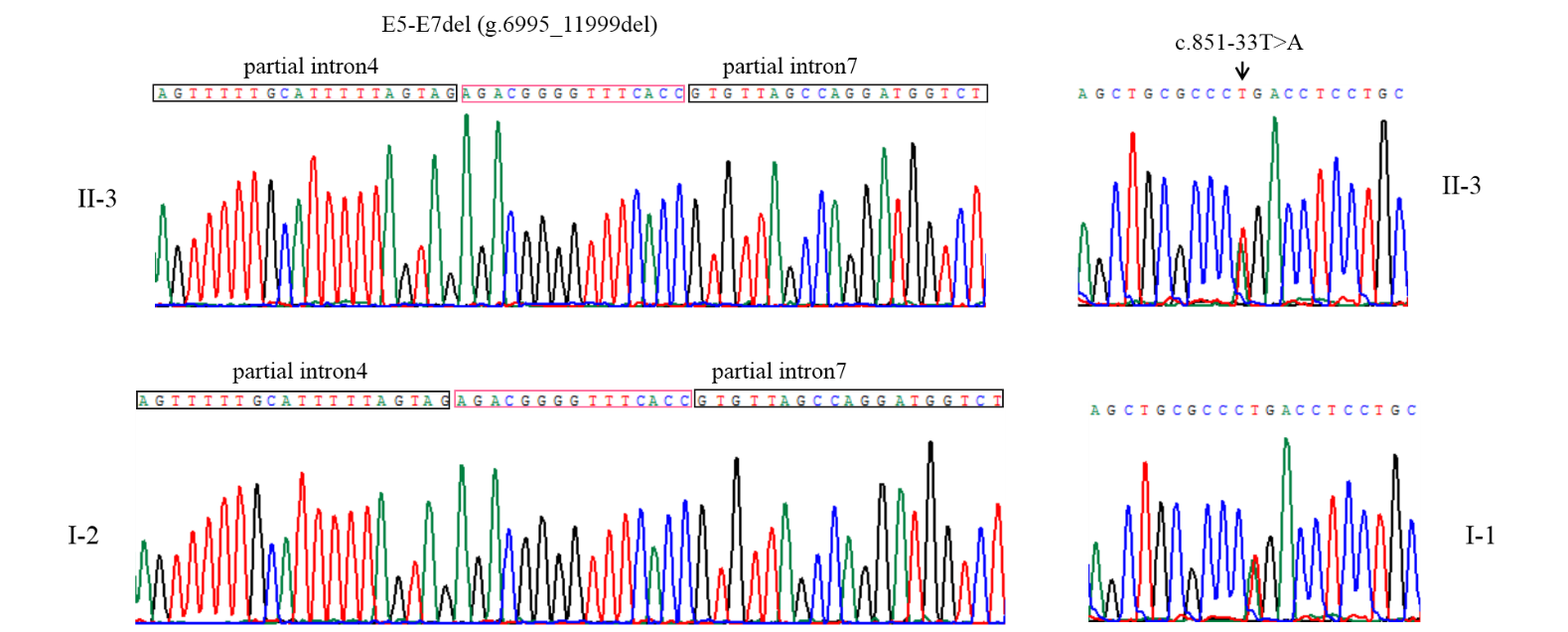


**Figure S1.Sequencing results of the family2.** The proband(II-3) carries heterozygous gross deletion: E6-E7del (g.6995_11999del) derived from the mother. The heterozygous splicing mutation of *NTRK1* gene c.851-33T>A was derived from the father.

**
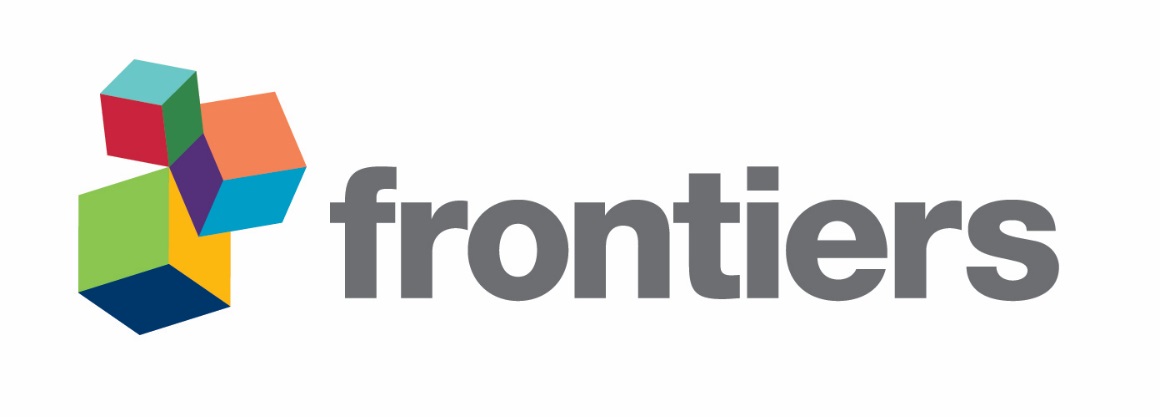
**
